# Does unwanted pregnancy lead to adverse health and healthcare utilization for mother and child? Evidence from low- and middle-income countries

**DOI:** 10.1007/s00038-020-01358-7

**Published:** 2020-04-09

**Authors:** Mohammad Hajizadeh, Son Nghiem

**Affiliations:** 1 School of Health Administration, Faculty of Health, Dalhousie University, Sir Charles Tupper Medical Building, 5850 College Street, 2nd Floor, Halifax, NS B3H 4R2 Canada; 2 The Centre for Applied Health Economics, Griffith University, Brisbane, Australia

**Keywords:** Unwanted pregnancy, Maternal and child healthcare, Child health, Low- and middle-income countries

## Abstract

**Objectives:**

Unwanted pregnancy is an important public health concern in low- and middle-income countries (LMICs). Using a pooled dataset from 48 Demographic Health Surveys conducted in Africa, Asia, Latin America and Europe (*n* = 494,778), we examined the effect of unwanted pregnancy on maternal and child healthcare utilization and child health outcomes in LMICs.

**Methods:**

We used logistic regression models to estimate the effect of unwanted pregnancy on antenatal care use, supervised delivery, childhood vaccination and three indicators of child health, viz. stunting (height-for-age), underweight (weight-for-age) and wasting (weight-for-height).

**Results:**

We found that mothers of children whose pregnancies had been unwanted had a lower probability of attending four or more antenatal care visits by 3.6% (95% confidence interval = 1.9–5.4%) compared to those whose pregnancy was wanted. We did not find significant impacts of unwanted pregnancy on supervised delivery, childhood vaccination uptake or child health indicators.

**Conclusions:**

Birth characteristics, household-level determinants and country-level characteristics seem to be more closely related to maternal and child healthcare utilization as well as child health outcomes than whether the pregnancy was wanted or unwanted in LMICs.

**Electronic supplementary material:**

The online version of this article (10.1007/s00038-020-01358-7) contains supplementary material, which is available to authorized users.

## Introduction

Globally, there are about 210 million pregnancies each year (World Health Organization 2012). Approximately 40% of these pregnancies are unintended (including mistimed or unwanted at the time of conception), and of these, 50% are aborted, 13% are miscarried and 38% are carried to term (Sedgh et al. 2014). The proportion of unintended pregnancy was found to be specifically higher among younger women (aged 15–19 years old) compared to older women (Ikamari et al. 2013). A pregnancy can be unintended for many reasons such as believing to have too many children already, no desire to have children at the moment, not having the financial resources available to support a new child, being in school, being unmarried, or the pregnancy might have been a result of incorrectly using or lack of access to contraceptives (Singh et al. 2006; Haffejee et al. 2018).

Unintended pregnancies can have significant health, social and economic impacts on the mother and her family (Singh et al. 2010). These can include negative effects on the physical and mental health of mothers as well as their quality of life (Schwarz et al. 2008; Khajehpour et al. 2013). In many low- and middle-income countries (LMICs), obtaining an abortion can be illegal or inaccessible, leading to many unsafe abortions either self-induced or performed by untrained professionals (Singh et al. 2018). Regardless of the method, abortions increase costs to the healthcare system and society through the costs associated with performing the abortion and treating complications as a result of a self-induced or unsafe abortion (Sonfield and Kost 2015). The health and social consequences of unintended pregnancies are significant in LMICs, where the majority of unintended pregnancies occur (Gipson et al. 2008).

If the woman cannot obtain an abortion, the unintended pregnancy can impact on her attitude and behaviour during pregnancy and her relationship with her child after it is born. It has been shown that unintended pregnancies are associated with delayed initiation of antenatal care, higher rates of maternal, neonatal and infant mortality and child nutrition status (Rahman 2015; Yazdkhasti et al. 2015). Long-term consequences for the unintended child include increased risk of cognitive impairment and chronic disease, reduced stature (Hoddinott et al. 2013) and an increased likelihood of crime in adulthood (Donohue and Levitt 2001).

To date, some studies (e.g. Marston and Cleland 2003; Wado et al. 2013; Rahman 2015; Singh et al. 2015; Rahman et al. 2016; Baschieri et al. 2017; Echaiz et al. 2018) have analysed the effect of unintended pregnancies on health and healthcare utilization in LMICs. However, the findings from these studies have been inconsistent. Two recent systematic reviews (Gipson et al. 2008; Hall et al. 2017) have called for more studies investigating unintended pregnancies in LMICs, given the limited number of studies and mixed results in the existing literature. Thus, using a dataset pooled from 48 Demographic and Health Surveys (DHS) conducted in Africa, Asia, Latin America and Europe linked with country-level indicators from the World Bank’s World Development Indicators and Global Development Finance (WDI and GDF) data sets (World Bank 2019), we analysed the impact of unwanted pregnancy on the receipt of antenatal care use, supervised delivery, childhood vaccination and child nutritional status as measured through stunting, underweight and wasting.

## Methods

### Data

The data for the analysis were obtained from DHS (https://www.dhsprogram.com/) collected from 48 LMICs through the Monitoring and Evaluation to Assess and Use Results the Standard Demographic and Health Surveys (MEASURE DHS) project over the period between 2010 and 2016. The standard DHS surveys are large nationally representative cross-sectional household surveys between 5000 and 30,000 households, typically conducted every 5 years in selected LMICs (The DHS Program 2019). The DHS collects comparable and reliable information on a variety of maternal and child health and healthcare indicators (Rutstein and Rojas 2006) by using a multistage sampling procedure (Demographic and Health Survey 1996). The data were collected using face-to-face interviews by trained interviewers. The DHS surveys use a similar set of questions to increase comparability across time and countries (Demographic and Health Survey 2006). Data collection methods and reliability and validation assessments can be found elsewhere (Rutstein and Rojas 2006). The final sample contained 494,778 singleton live births, aged 1–59 months, from 48 LMICs. Selection of countries was determined by the availability of DHS surveys between 2010 and 2016. In addition, the World Bank’s WDI and GDF datasets (World Bank 2019) were used to obtain country-level information. The country-level information was linked to each child included in the DHS surveys using the child’s birth year. Table 1 reports survey years, sample size and gross domestic product (GDP) per capita for the sampled countries.

**Table 1:** Survey year, sample size and gross domestic product per capita for the sampled 48 low- and middle-income countries from Africa, Asia, Latin America and Europe, Demographic Health Surveys, 2010–2016

Country	Country code	Survey year	Sample size		Gross domestic product per capita^a^
			Household	Children 0–59 months	
*Low*-*income countries*^b^					
Afghanistan	AF	2015	24,395	32,026	1808
Bangladesh	BD	2014	17,300	5381	2979
Benin	BJ	2011–2012	17,422	12,712	1779
Burkina Faso	BF	2010	14,424	14,455	1421
Burundi	BI	2010	8596	7558	708
Cambodia	KH	2014	15,825	7029	3113
Chad	TD	2014–2015	17,233	18,024	2059
Comoros	KM	2012	4482	3000	1403
Congo Democratic Republic	CD	2013–2014	18,171	18,010	692
Gambia	GM	2013	6217	7795	1593
Guinea	GN	2012	7109	6720	1197
Haiti	HT	2012	13,181	7025	1585
Liberia	LR	2013	9333	7300	817
Malawi	MW	2010	24,825	19,093	1061
Mali	ML	2012–2013	10,105	9982	1787
Mozambique	MZ	2011	13,919	10,657	952
Nepal	NP	2011	10,826	5240	2042
Niger	NE	2012	10,750	12,125	867
Rwanda	RW	2014–2015	12,699	7629	1620
Sierra Leone	SL	2013	12,629	11,411	1854
Tajikistan	TJ	2012	6432	4889	2343
Tanzania	TZ	2015–2016	12,563	9780	2510
Togo	TG	2013–2014	9549	6670	1316
Uganda	UG	2011	9033	7621	1665
Zimbabwe	ZW	2010–2011	9756	5416	1456
*Lower*-*middle*-*income countries*^b^					
Armenia	AM	2010	6700	1453	6508
Cameroon	CM	2011	14,214	11,156	2614
Congo Brazzaville	CG	2011–2012	11,632	8927	5665
Cote d’Ivoire	CI	2011–2012	9686	7402	2650
Egypt	EG	2014	28,175	15,227	10,049
Ghana	GH	2014	11,835	5597	3894
Honduras	HN	2011–2012	21,362	10,719	4491
Indonesia	ID	2012	43,852	17,672	9283
Kenya	KE	2014	36,430	9811	2819
Kyrgyz Republic	KG	2012	8040	4267	2870
Lesotho	LS	2014	9402	3051	2760
Nigeria	NG	2013	38,522	30,252	5448
Pakistan	PK	2012–2013	12,943	11,483	4429
Philippines	PH	2013	14,804	7099	6366
Senegal	SN	2014	4231	13,122	2202
Yemen	YE	2013	17,351	15,731	3879
Zambia	ZM	2013–2014	15,920	13,020	3582
*Upper*-*middle*-*income countries*^b^					
Colombia	CO	2010	51,447	17,487	10,901
Dominican Republic	DO	2013	11,464	3628	11,888
Gabon	GA	2012	9755	5787	17,595
Jordan	JO	2012	15,190	9993	10,243
Namibia	NA	2013	9849	4892	9140
Peru	PE	2012	27,218	9454	10,944
Total			746,796	494,778	

^a^The gross domestic product per capita at purchasing power parity, constant 2011 international $

^b^The 2017 World Bank classification of the world’s economies is used to categorize sampled countries into low-income countries, lower–middle-income countries and upper–middle-income countries groups

### Variables

#### Outcome variables

We examined the effect of unwanted pregnancy carried to term (hereafter ‘unwanted pregnancy’) on antenatal care use, supervised delivery and childhood vaccination as outcome measures of healthcare utilization during the antenatal/prenatal, intranatal and postpartum/postnatal stages, respectively. In addition, we looked at the effect of unwanted pregnancy on three child health outcome indicators: stunting (height-for-age); underweight (weight-for-age); and wasting (weight-for-height). These outcomes were chosen based on previous studies (Marston and Cleland 2003) to reflect the different stages at which an unwanted pregnancy might have an impact on health outcomes.

The 2016 World Health Organization (WHO) guideline of antenatal care (ANC) recommends a minimum of eight contacts to reduce perinatal mortality and improve mother’s experience of care. As our analyses used data before the change in the guideline, we used the 2006 WHO recommendation (World Health Organization 2006a) and defined adequate ANC use as a binary variable indicating whether or not the mother attended four or more ANC visits during her pregnancy. Supervised delivery was measured as a binary variable representing whether or not health professionals (e.g. a midwife, doctor or nurse) assisted the delivery, as per the WHO definition (World Health Organization 2004). Childhood vaccination was measured by a binary variable of whether or not the child completed the WHO recommended immunization schedule (see Table A.1 in the Online Resource) for four routine vaccines, viz. Bacillus Calmette–Guérin (BCG), polio (3 doses), diphtheria, tetanus and pertussis (DTP, 3 doses) and measles vaccines (World Health Organization 2019). Children were considered as vaccinated if they were younger than 11 months and completed the WHO recommended immunization schedule or they were 11 months and older and completed all the four routine vaccines.

Three binary variables were used to measure child health outcome. Childhood stunting was defined whether or not the height-for-age *Z*-score (HAZ) of the child was below two standard deviations (HAZ < − 2SD) from the median of the reference population as defined by the WHO growth standards (World Health Organization 2006b). Similarly, childhood wasting and underweight were measured by whether or not the weight-for-height *Z*-score (WHZ) and weight-for-age *Z*-score (WAZ) of the child were below two SD from the median of the reference population as defined by the WHO growth standards, respectively (World Health Organization 2006b). These analyses were restricted to surviving children with HAZ, WHZ and WAZ values between − 6 and 6 as *Z*-scores outside this range are biologically implausible values based on WHO definition (De Onis 2006). We generated *Z*-scores for height-for-age, weight-for-height and weight-for-age using the 2006 WHO growth standard (World Health Organization 2006b) and the Stata’s user-written programme *zscore06* (Leroy 2011).

#### Exposure variable

Whether a pregnancy was wanted or not was determined based on the intention status of the pregnancy: wanted then, wanted later, not wanted. The intention status of the pregnancy was collected using the following question in the DHS enquiries: “At the time you became pregnant with (name of child), did you want to become pregnant then, did you want to wait until later, or did you want no more children at all?” (Marston and Cleland 2003). Previous studies have often used wanted/planned, mistimed and unwanted terms, respectively, to represent the intention status of the pregnancies (e.g. Marston and Cleland 2003; D’Angelo et al. 2004). Since mistimed pregnancies are ultimately wanted pregnancies and studies (Marston and Cleland 2003) have documented non-significant or reduced differences between mistimed and wanted pregnancies, in this study, as other studies have previously done (e.g. Barrick and Koenig 2008), we classified mistimed pregnancies as wanted pregnancies.

#### Control variables

Based on the extant literature (Marston and Cleland 2003; Hajizadeh et al. 2015; Rahman 2015; Singh et al. 2015; Rahman et al. 2016; Baschieri et al. 2017; Hajizadeh 2019), we controlled for birth characteristics (gender, age of child, birth order) and household-level covariates (mother’s age at birth, mother’s marital status, mother’s education, household living standard/wealth and region) that have been consistently collected in all DHS surveys and country-level covariates [GDP per capita and public health expenditures per capita in purchasing power parity (PPP), 2011 prices, international $] in our analysis. A constructed wealth index (WI) for each household in the DHS surveys was used as a measure of the household living standard. Using a method suggested by Filmer and Pritchett (2001), the DHS uses information collected on selected household’s assets (e.g. bicycles, radio, televisions), types of sanitation facilities, water source and building materials to construct a measure of household living standard, the WI (Rutstein and Johnson 2004). We used the WDI and GDF data sets (World Bank 2019) to obtain country-level covariates for each country in our study. To correct for excessive skewness, we used the natural logarithm transformation of GDP per capita and public health expenditures per capita in the analysis. Table 2 reports the definitions and summary statistics of all the variables used in the study.

**Table 2:** Definitions and summary statistics of variables used in the analysis, Demographic Health Surveys from 48 low- and middle-income countries in Africa, Asia, Latin America and Europe, 2010–2016

Variable	Definition	Mean	SD
*Outcome variables*			
Antenatal care	= 1 if mother received four or more antenatal care, 0 otherwise	0.58	0.49
Supervised delivery	= 1 if delivery assisted by a health professional such as a midwife, doctor or nurse. 0 otherwise	0.52	0.50
Child vaccination	= 1 if the child completed the WHO recommended immunization schedule for four routine vaccines, 0 otherwise	0.49	0.50
Stunting	= 1 if the height-for-age Z-score (HAZ) of the child is below minus two SD from the median of the reference population, defined by the WHO growth standards, 0 otherwise	0.33	0.47
Underweight	= 1 if the weight-for-height Z-Score (WHZ) of the child is below minus two SD from the median of the reference population, defined by the WHO growth standards, 0 otherwise	0.21	0.41
Wasting	= 1 if the weight-for-age (WAZ) of the child is below minus two SD from the median of the reference population, defined by the WHO growth standards, 0 otherwise	0.11	0.31
*Exposure variable*			
Unwanted pregnancy	= 1 if the pregnancy is unwanted, 0 otherwise	0.09	0.28
Wanted pregnancy (Ref.)	= 1 if the pregnancy is wanted, 0 otherwise	0.91	0.28
*Birth characteristics*			
Male	= 1 if the child is male, 0 otherwise	0.51	0.50
Female (Ref.)	= 1 if the child is female, 0 otherwise	0.49	0.50
Age of child (years)	Child’s age in years	2.40	1.43
Birth order# 1 (Ref.)	= 1 if the birth order of the child is one, 0 otherwise	0.26	0.44
Birth order# 2	= 1 if the birth order of the child is two, 0 otherwise	0.22	0.41
Birth order# 3 and above	= 1 if the birth order of the child is three or above, 0 otherwise	0.51	0.50
*Household*-*level covariates*			
Mother’s age at birth (< 20 years)	= 1 if the mother’s age at birth is less than 20 years, 0 otherwise	0.10	0.30
Mother’s age at birth (19 < years < 41) (Ref.)	= 1 if the mother’s age at birth is between 20 and 40 years, 0 otherwise	0.85	0.36
Mother’s age at birth (> 40 years)	= 1 if the mother’s age at birth is greater than 40 years, 0 otherwise	0.05	0.21
Mother’s marital status—married (Ref.)	= 1 if the mother is married, 0 otherwise, 0 otherwise	0.84	0.37
Mother’s marital status—formally married	= 1 if the mother is formally married, 0 otherwise	0.16	0.37
Mother’s education (years)	Mother’s education in years	5.38	4.94
Household socio-economic status, 1st quintile (Ref.)	1 = if the socio-economic status of the household is in the first (lowest) quintile, 0 otherwise	0.20	0.40
Household socio-economic status, 2nd quintile	1 = if the socio-economic status of the household is in the second quintile, 0 otherwise	0.20	0.40
Household socio-economic status, 3rd quintile	1 = if the socio-economic status of the household is in the third quintile, 0 otherwise	0.20	0.40
Household socio-economic status, 4th quintile	1 = if the socio-economic status of the household is in the fourth quintile, 0 otherwise	0.20	0.40
Household socio-economic status, 5th quintile	1 = if the socio-economic status of the household is in the fifth (highest) quintile, 0 otherwise	0.20	0.40
Rural	1 = if the child resides in a rural area, 0 otherwise	0.67	0.47
Urban (Ref.)	1 = if the child resides in an urban area, 0 otherwise	0.33	0.47
*Country*-*level covariates*			
Log gross domestic product per capita	Natural log of gross domestic product per capita at purchasing power parity, constant 2011 international $	8.11	0.79
Log public health expenditures per capita	Natural log of public health expenditures per capita at purchasing power parity, constant 2011 international $	3.95	0.82
*Country fixed effects covariates*			
48 dummy variables for countries	= 1 if the child was born in the country, 0 otherwise (dummy variable for Nigeria was used as Ref. )	–	–

We used the de-normalized standard weight (as per the Demographic Health Survey Sampling and Household Listing Manual (ICF International 2012) as a weight to compute summary statistics

*SD* standard deviation, *WHO* World Health Organization, Ref. reference category in the regression analyses

### Statistical analysis

Since all outcome variables were binary, we used multivariable logistic regressions to examine the extent to which unwanted pregnancy affects ANC, supervised delivery, childhood vaccination and child health outcomes, controlling for exogenous independent variables. A general specification of the multivariable logistic regression is:

1 $${\text{Log}}\,it\left[ {\pi \left( {y_{it} } \right)} \right] = \beta + \gamma x_{it} + \alpha_{i} + \varepsilon_{it},$$

where $\left( {y_{it} } \right) = \frac{p\left( y \right)}{1 + p\left( y \right)}$, which transforms the probability of outcome $y$ (e.g. stunting, wasting and childhood vaccination) of country $i$ in a period $t$, $p\left( y \right)$, from (0, 1) to (− ∞, + ∞), allowing a standard linear regression to be applied. $x$ are the set of independent variables [e.g. age, sex and socio-economic status (SES)] affecting the probability that outcome $y$ will occur, $\alpha$ represents country dummies (or fixed effects), and $\varepsilon$ is the random noise. We included county fixed effects to account for unobserved heterogeneity between countries (e.g. cultural differences) that effects our outcome variables. The parameters of interest (*γ*) are difficult to interpret directly. Thus, we calculated marginal effects at the means of the independent variables to report the effect of each explanatory variable on the probability of the outcome variables. Based on the annual female population provided by the Population Division of the United Nations (UN DESA 2020), we applied the de-normalization of standard weights approach, as per the DHS Sampling and Household Listing Manual (ICF International 2012), to calculate an appropriate weight for each observation in the analyses. We calculated 95% confidence intervals (CI) for the marginal effects, taking into account the effect of the geographical clustering of the sample. All analyses were performed with Stata software package (version 15, StataCorp, College Station, Tex).

## Results

### Unwanted pregnancy in LMICs

**Fig. 1:**
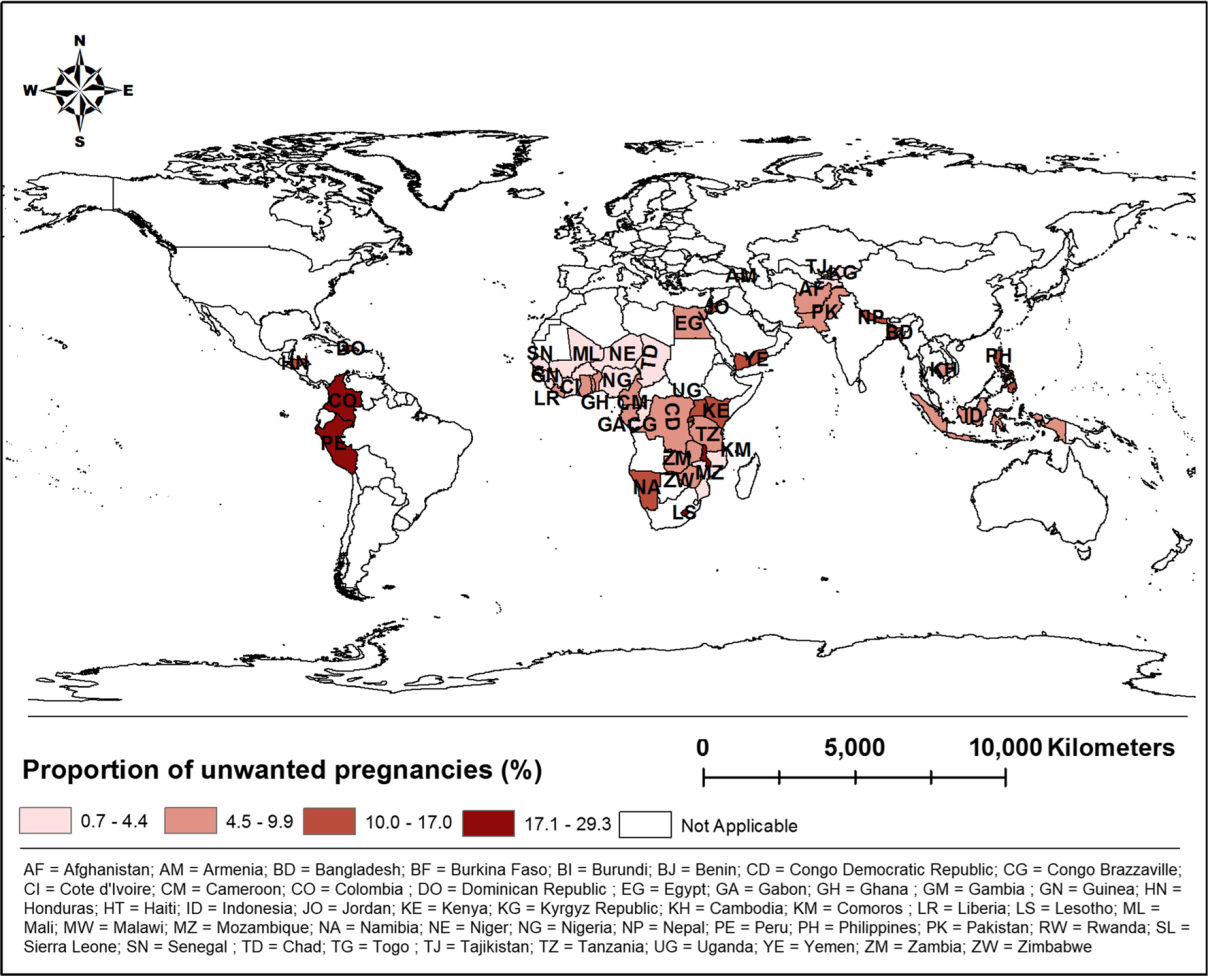
Proportion of unwanted pregnancies in 48 low- and middle-income countries from Africa, Asia, Latin America and Europe, Demographic Health Surveys, 2010–2016

Table A.2 in the Online Resource shows the proportion of pregnancies (%) reported as unwanted in the 48 LMICs by sex and region. The overall prevalence of unwanted pregnancies was 9% in the sampled countries. The proportion of pregnancies reported as unwanted in the 48 LMICs varied from less than 1% in the Kyrgyz Republic to about 30% in Malawi (see Table A.2 in the Online Resource and Fig. 1). The proportion of unwanted pregnancies for male and female was 8.54% and 8.58%, respectively. The proportion of unwanted pregnancies was 9.27% in rural areas, whereas this figure was 8.21% in urban areas.

**Fig. 2:**
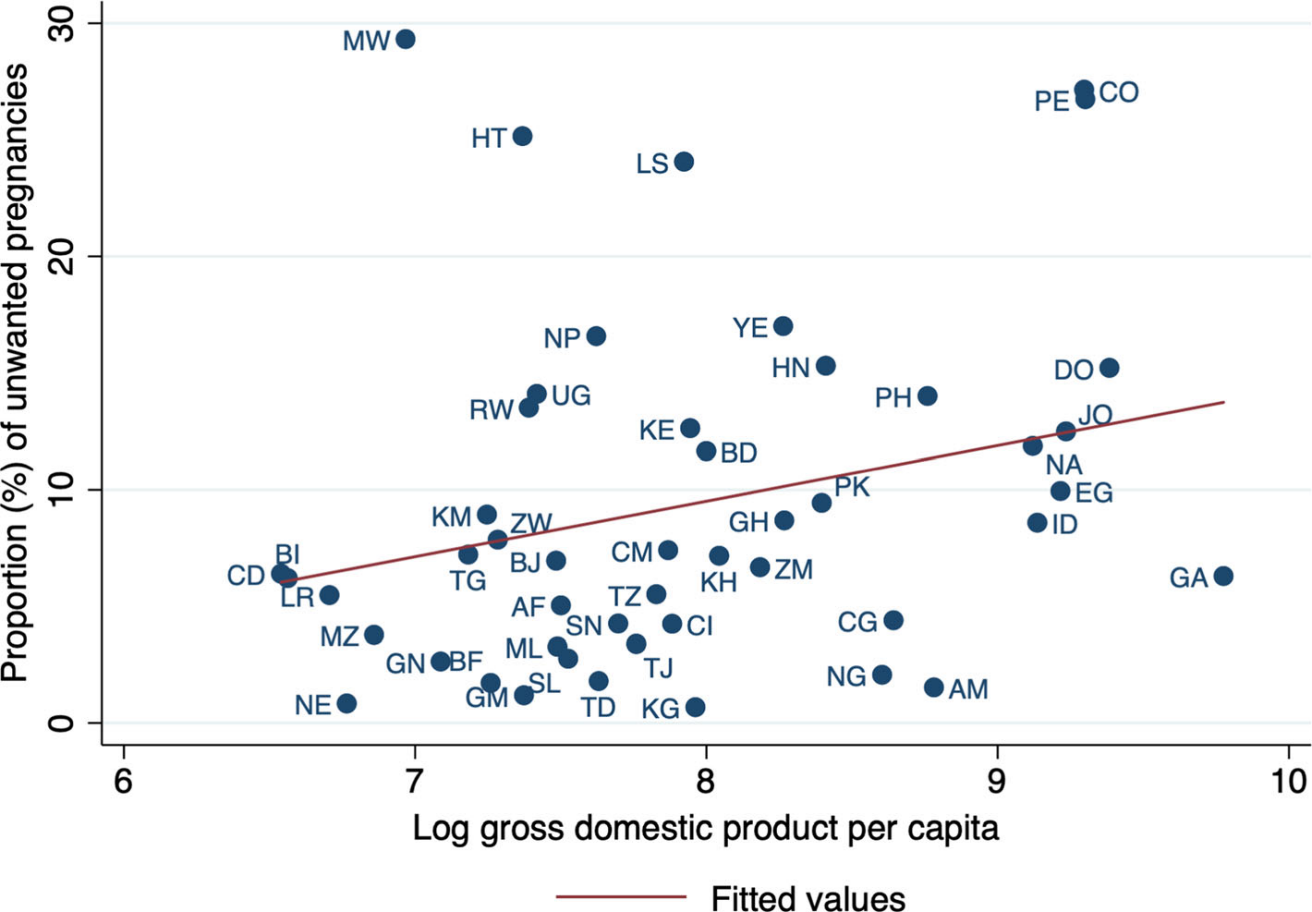
Cross-country correlation between the proportion (%) of unwanted pregnancies and log gross domestic product per capita in 48 low- and middle-income countries from Africa, Asia, Latin America and Europe, Demographic Health Surveys, 2010–2016

Figure 2 shows the cross-country correlation between the proportion of unwanted pregnancies and log GDP per capita. There was a positive but weak association (beta = 2.4, *p* value = 0.07) between the log of GDP per capita and the log of the proportion of unwanted pregnancies across countries: a 1% increase in GDP per capita was associated with 0.024% increase in the proportion of unwanted pregnancy.

### Unwanted pregnancy and maternal/child healthcare and child health outcomes

Table 3 provides the results of multivariable logistic regression, showing the relationship between unwanted pregnancy and maternal and child healthcare and child health outcomes in the 48 LMICs after adjusting for the potential confounding factors, including birth characteristics and household-level covariates, country-level covariates and county fixed effects. Results indicated that mothers whose pregnancy was unwanted had a lower probability of having adequate ANC by 3.6% [95% confidence interval (CI) 1.9–5.4%] compared to those whose pregnancy was wanted. There were no significant differences in supervised delivery, childhood vaccination and nutritional status (measured by stunting, underweight and wasting) of children born from unwanted and wanted pregnancies.

**Table 3:** The relationship between unwanted pregnancy and maternal and child healthcare and child health outcomes in 48 low- and middle-income countries from Africa, Asia, Latin America and Europe, Demographic Health Surveys, 2010–2016

	Antenatal stage	Intranatal stage	Postpartum stage	Health outcomes		
	Antenatal care^a^	Supervised delivery	Vaccination	Stunting^b^	Underweight^b^	Wasting^b^
*Exposure variable*						
Unwanted pregnancy	− 3.6***	0.6	1.4*	− 1.1	− 1.3*	− 0.5
	(− 5.4 to − 1.9)	(− 1.2 to 2.4)	(− 0.1 to 2.9)	(− 2.7 to 0.5)	(− 2.7 to 0.1)	(− 1.6 to 0.6)
Wanted birth (Ref.)						
*Birth characteristics*						
Male	1***	1.6***	0.2	4.4***	2.6***	1***
	(0.3 to 1.7)	(0.9 to 2.3)	(− 0.4 to 0.8)	(3.6 to 5.1)	(2 to 3.2)	(0.6 to 1.5)
Female (Ref.)						
Age of child (years)	0.1	− 2.5***	2.9***	3.8***	0.7***	− 1.9***
	(− 0.3 to 0.5)	(− 2.8 to − 2.1)	(2.6 to 3.3)	(3.5 to 4.1)	(0.4 to 1)	(− 2.1 to − 1.7)
Birth order# 1 (Ref.)						
Birth order # 2	− 6.4***	− 7.9***	− 1.3***	1.9***	0.6	0.1
	(− 7.6 to − 5.1)	(− 9 to − 6.8)	(− 2.2 to − 0.3)	(0.9 to 3)	(− 0.5 to 1.7)	(− 0.5 to 0.8)
Birth order # 3 and above	− 9.7***	− 13.2***	− 3.5***	1.8***	1.3***	0.1
	(− 10.9 to − 8.5)	(− 14.4 to − 11.9)	(− 4.5 to − 2.5)	(0.7 to 2.9)	(0.4 to 2.3)	(− 0.5 to 0.7)
*Household*-*level covariate*						
Mother’s age at birth (< 20 years)	− 8.2***	− 6.1***	− 6.7***	5.9***	3.5***	0.8*
	(− 9.7 to − 6.8)	(− 7.6 to − 4.6)	(− 7.8 to − 5.5)	(4.4 to 7.4)	(2.3 to 4.6)	(0.1 to 1.6)
Mother’s age at birth (19 < years < 41) (Ref.)						
Mother’s age at birth (> 40 years)	0.7	4.7***	− 0.8	− 1.4*	− 0.9	0.3
	(− 0.6 to 2.1)	(2.9 to 6.6)	(− 2.4 to 0.8)	(− 3 to 0.2)	(− 2.2 to 0.4)	(− 0.6 to 1.1)
Mother’s marital status (formally married)	− 4.8***	2.3***	− 2.6***	0.1	− 0.7	− 0.4
	(− 5.9 to − 3.6)	(0.8 to 3.7)	(− 3.6 to − 1.6)	(− 0.9 to 1.1)	(− 1.5 to 0.2)	(− 0.9 to 0.2)
Mother’s education (years)	2.5***	2.7***	1.7***	− 1.1***	− 0.8***	− 0.2***
	(2.3 to 2.6)	(2.6 to 2.9)	(1.6 to 1.9)	(− 1.3 to − 1)	(− 0.9 to − 0.7)	(− 0.3 to − 0.2)
Household socio-economic status, 1st quintile (Ref.)						
Household socio-economic status, 2nd quintile	7***	9***	7.9***	− 3.5***	− 3.4***	− 1.1***
	(5.8 to 8.2)	(7.2 to 10.7)	(6.4 to 9.3)	(− 4.7 to − 2.3)	(− 4.4 to − 2.3)	(− 1.9 to − 0.4)
Household socio-economic status, 3rd quintile	12.4***	17.4***	11.4***	− 6.9***	− 5.7***	− 1.7***
	(11.1 to 13.8)	(15.4 to 19.4)	(9.8 to 13)	(− 8.3 to − 5.6)	(− 6.9 to − 4.6)	(− 2.6 to − 0.9)
Household socio-economic status, 4th quintile	19.1***	27.2***	15***	− 10.8***	− 8.4***	− 1.9***
	(17.6 to 20.6)	(25 to 29.3)	(13.3 to 16.8)	(− 12.3 to − 9.3)	(− 9.6 to − 7.2)	(− 2.8 to − 0.9)
Household socio-economic status, 5th quintile (highest)	28.2***	39.4***	18.8***	− 17.5***	− 12.7***	− 3.1***
	(26.4 to 30.1)	(37 to 41.8)	(16.9 to 20.6)	(− 19.4 to − 15.6)	(− 14.2 to − 11.2)	(− 4.1 to − 2.1)
Rural	− 5.4***	− 11.1***	0.2	− 0.9	− 1.6**	− 1**
	(− 6.8 to − 4)	(− 12.9 to − 9.2)	(− 1.2 to 1.7)	(− 2.2 to 0.5)	(− 2.7 to − 0.4)	(− 1.9 to − 0.2)
Urban (Ref.)						
*Country*-*level covariates*						
Log gross domestic product per capita	− 24.9***	4.8***	16.6***	− 13.1***	− 14.8***	0.6
	(− 35.7 to − 14.1)	(4.4 to 14)	(7.3 to 25.9)	(− 22 to − 4.3)	(− 22.9 to − 6.7)	(− 5.2 to 6.4)
Log public health expenditures per capita	− 1	3.4***	− 4.7***	− 5.4***	− 3***	0.8
	(− 2.8 to 0.8)	(2.1 to 4.7)	(− 6 to − 3.3)	(− 6.9 to − 3.9)	(− 4.2 to − 1.7)	(− 0.2 to 1.8)
*Pseudo-R*-*square*	0.256	0.272	0.121	0.081	0.111	0.075
*N*	346,502	494,584	480,759	275,571	282,953	273,835

All regressions include country dummies with Nigeria as reference category

Reported estimates are marginal effects calculated at the means of the independent variable. The reported marginal effects are multiplied by 100 for readability

The values in parentheses are 95% confidence intervals

Ref. = Reference category in the regression analyses

Significance: ***p < 0.01, **p < 0.05, *p < 0.1

^a^We performed our analysis on the number of antenatal care visits. Results indicated that mothers of children with unwanted pregnancies, on average, used − 0.12 (95% confidence interval: − 0.21 to − 0.03) less antenatal care visits than those with wanted pregnancies

^b^Stunting, underweight and wasting data were unavailable for Afghanistan, Indonesia and Philippines, and observations from these countries were therefore dropped from the analysis

The results revealed significant effects of birth characteristics, household-level determinants and country-level characteristics on maternal and child healthcare utilization, as well as child health outcomes. In particular, compared with girls, boys were 1% (95% CI 0.3–0.5%) more likely to receive prenatal care or 1.6% (95% CI 0.9–2.3%) more likely to receive supervised delivery. The probability of stunting, underweight or wasting in boys was higher than that of girls by 4.4% (95% CI 3.6–5.1%), 2.6% (95% CI 2–3.2%) and 1% (95% CI: 0.6 to 1.5%), respectively. Birth order was one of the most influential factors contributing to prenatal care, intranatal care, postnatal care and health outcomes. Compared with children of birth order one (i.e. the first child), children of birth order two and above received lower antenatal care, supervised delivery and childhood vaccination. Mother’s age at birth also played a critical role in pregnancy care and child health. Children to mothers who were younger than 20 years old (compared with 20 and 40 years old) at the time of birth had a significantly lower probability of antenatal care, supervised delivery and childhood vaccination, while having a higher probability of stunting and underweight. Mother’s education and household SES had significant positive effects on pregnancy care and child health. The positive effects of SES were also present at the country level, where higher GDP per capita and public health expenditure were associated with a reduction in stunting by and underweight among children.

## Discussion

Unwanted pregnancy is an important public health problem in LMICs countries, especially among young women (Singh and Darroch 2000). Notwithstanding the high prevalence of unwanted pregnancies in LMICs, there has been a limited number of studies investigating the relationship between unwanted pregnancies and maternal and child healthcare utilization and child health outcomes. We used a large dataset pooled from 48 Demographic Health Surveys (DHS) conducted in Africa, Asia, Latin America and Europe to examine the effect of unwanted pregnancy on maternal and child healthcare utilization and child health outcomes.

A key finding of our study was that unwanted pregnancy reduced the receipt of adequate ANC (at least four visits). This result is consistent to those found in Bangladesh and Nepal where unwanted pregnancy was associated with the delay in receiving antenatal care and attending adequate ANC (at least four visits) (Singh et al. 2015; Rahman et al. 2016). This trend continued in some sub-Saharan Africa countries such as Kenya (Ochako and Gichuhi 2016) and Nigeria, where unwanted pregnancies were associated with late and fewer antenatal care visits (Amo-Adjei and Anamaale Tuoyire 2016). Unwanted pregnancies may reduce the receipt of antenatal care for various reasons. For example, if the woman does not recognize she is pregnant, does not want to acknowledge her pregnancy, or does not want others to know (which can be the case if the pregnancy was the result of rape or incest), she may not seek antenatal care (Rahman et al. 2016).

Although some studies (Marston and Cleland 2003; Rahman et al. 2016) have indicated that whether pregnancy wanted or not affects supervised delivery, we did not find any significant difference in supervised delivery of children born from wanted and unwanted pregnancies. Findings in the literature have also been inconsistent; in Bangladesh and Peru, women with unwanted pregnancies were less likely to pursue professional delivery services (Marston and Cleland 2003; Rahman et al. 2016), but a similar trend was not present in Egypt, Kenya, Bolivia, the Philippines (Marston and Cleland 2003) and Ethiopia (Wado et al. 2013). Apart from the attitude towards the unwanted pregnancy of an expecting mother, her financial position may influence the decision to have supervised delivery or not (Rahman et al. 2016). The inconsistency between the results of our study and the findings from Peru (Marston and Cleland 2003) could be due to the difference in the study population and time frame of the studies. Differences in the population and definition of supervised delivery may explain the discrepancy between our result and the result of the previous study from Bangladesh (Rahman et al. 2016).

Despite the potential negative impact of unwanted pregnancies on child health outcomes, thought to be due to conscious or unconscious maternal feelings and behaviours towards the unwanted pregnancy leading to neglect (Singh et al. 2017), we did not find such an effect in this study. Our results did not reveal a significant effect of unwanted pregnancy on childhood vaccination or in child nutritional status measured by stunting, underweight and wasting. In the literature, evidence for the association between unwanted pregnancy and childhood vaccination and child nutritional status has been variable. Studies in Nepal, Kenya and Peru have found unwanted pregnancies to be associated with incomplete vaccination statuses by the child’s first birthday (Marston and Cleland 2003; Singh et al. 2015; Echaiz et al. 2018), but no relationship was found in Bolivia and the Philippines (Marston and Cleland 2003). Likewise, for nutritional status, children of unwanted pregnancies in Bangladesh were more likely to be stunted, wasted and underweight (Rahman 2015); however, no effect was found between pregnancy intention and stunting in Malawi (Baschieri et al. 2017). The inconsistency between our results and the previous findings could be explained by the differences in the study population and time frame of the studies.

The key strength of this study that enriches the current literature was the use of a large representative sample drawn from 48 LMICs countries. The large sample size enabled us to have sufficient power to assess adverse health and healthcare utilization for mother and child. Using the large pooled dataset helped us to improve the previous findings reported for a single country. There are, however, some limitations to this study. First, self-report of unwanted pregnancy is subject to criticism as it is self-reported by mothers after children are born and thus may be subject to recall bias. Second, as the DHS collects data on living children at the time of the survey, the results of this study indicated the impact of unwanted pregnancy on children who were alive at the time of the survey, discounting any effect of unwanted pregnancy on children who died prior to the survey. This may underestimate the adverse impact of unwanted pregnancy on health and healthcare utilization for mother and child if unwanted pregnancy positively associated with child mortality in LMICs. Third, some of the time-varying covariates in the study are subject to measurement error (e.g. the household’s SES) because they are reported at the time of interview and assigned to all prior births. Fourth, other factors such as family and social environments and health system characteristics may also influence maternal and child health and healthcare, but were excluded from our analyses because of lack of information in DHS. Fifth, because of the cross-sectional design of the study, it was not possible to establish temporality; thus, the evidence for causality can only be suggested. Finally, with the use of sampling weights in the analysis, the results of this study are generalizable only to the 48 LMICs included in our analyses and further extrapolation of the findings to other countries should be done with caution.

### Conclusion

Although we found an adverse impact of unwanted pregnancy on antennal care, our result did not suggest any association between unwanted pregnancy on supervised delivery, childhood vaccination and nutritional status of children. Our results indicated that birth characteristics, household-level determinants and country-level characteristics seem to be more closely related to maternal and child healthcare utilization as well as nutritional status of children than whether the pregnancy was wanted or not in LMICs.

## Electronic supplementary material

Below is the link to the electronic supplementary material.

Supplementary material 1 (DOCX 34 kb)
