# Supplementary material for: Does unwanted pregnancy lead to adverse health and healthcare utilization for mother and child? Evidence from low- and middle-income countries
Source: Int J Public Health. 2020 Apr 9;65(4):457–68. doi: 10.1007/s00038-020-01358-7 (PMC7275006; doi:10.1007/s00038-020-01358-7)
Supplement: Supplementary file 1 — Supplementary material 1 (DOCX 34 kb) [file 38_2020_1358_MOESM1_ESM.docx]

**Journal Name:** International Journal of Public Health

**Title:** Does unwanted pregnancy lead to adverse health and healthcare utilization for mother and child? Evidence from low- and middle-income countries

**Table A.1:** The WHO recommended immunization schedules for four core vaccines

| **Vaccine** | **Age of the first dose** | **Interval between doses** | |
| --- | --- | --- | --- |
|  |  | **First and second** | **Second and third** |
| **Bacillus Calmette-Guérin (BCG)** | As soon as possible after birth |  |  |
| **Diphtheria-tetanus-pertussis (DTP)**† | 6 weeks (Min‡) | 4 weeks (Min) | 4 weeks (Min) |
| **Polio** | 6 weeks (Min) | 4 weeks (Min) | 4 weeks (Min) |
| **Measles** | 39 (Min) or 52 Weeks [9 or 12 months] |  |  |

Adopted from: http://www.who.int/immunization/policy/Immunization_routine_table2.pdf?ua=1

† Polio 1 = bOPV + IPV, Polio 2 = IPV / bOPV Sequential, Polio 3 = IPV

‡ Min: Minimum

**Table A.2:** Percentage proportion of pregnancy reported unwanted in 48 low- and middle-income countries by sex and region, Demographic Health Surveys, 2010-2016

| **Country** | | **Total** | **Sex**† | | **Region**‡ | |
| --- | --- | --- | --- | --- | --- | --- |
|  |  |  | **Male** | **Female** | **Urban** | **Rural** |
| **Low-income countries** | | | | | | |
|  | Afghanistan | 5.04 | 5.09 | 5.00 | 7.87 | 4.22 |
|  | Bangladesh | 11.66 | 11.92 | 11.38 | 8.06 | 12.86 |
|  | Benin | 6.96 | 7.08 | 6.84 | 7.63 | 6.52 |
|  | Burkina Faso | 1.72 | 1.68 | 1.75 | 2.71 | 1.52 |
|  | Burundi | 6.21 | 6.29 | 6.12 | 7.64 | 6.08 |
|  | Cambodia | 7.17 | 6.88 | 7.47 | 5.42 | 7.46 |
|  | Chad | 1.80 | 1.72 | 1.89 | 2.83 | 1.55 |
|  | Comoros | 8.94 | 8.84 | 9.04 | 7.43 | 9.50 |
|  | Congo Democratic Republic | 6.40 | 6.31 | 6.48 | 9.53 | 5.02 |
|  | Gambia | 1.19 | 1.07 | 1.32 | 0.97 | 1.39 |
|  | Guinea | 2.64 | 2.57 | 2.71 | 3.39 | 2.37 |
|  | Haiti | 25.17 | 25.41 | 24.93 | 21.83 | 27.09 |
|  | Liberia | 5.48 | 5.36 | 5.60 | 4.55 | 6.41 |
|  | Malawi | 29.32 | 29.30 | 29.35 | 29.77 | 29.25 |
|  | Mali | 3.27 | 3.42 | 3.11 | 3.72 | 3.16 |
|  | Mozambique | 3.78 | 3.66 | 3.89 | 6.33 | 2.80 |
|  | Nepal | 16.59 | 14.61 | 18.71 | 10.24 | 17.25 |
|  | Niger | 0.84 | 0.89 | 0.79 | 1.48 | 0.75 |
|  | Rwanda | 13.52 | 13.50 | 13.54 | 12.94 | 13.64 |
|  | Sierra Leone | 2.76 | 2.51 | 3.01 | 4.23 | 2.26 |
|  | Tajikistan | 3.39 | 3.35 | 3.43 | 1.93 | 3.79 |
|  | Tanzania | 5.52 | 5.63 | 5.40 | 6.04 | 5.32 |
|  | Togo | 7.22 | 6.90 | 7.55 | 8.70 | 6.40 |
|  | Uganda | 14.11 | 13.52 | 14.71 | 9.43 | 14.88 |
|  | Zimbabwe | 7.86 | 7.73 | 7.99 | 7.54 | 7.99 |
| **Lower-middle-income countries** | | | | | | |
|  | Armenia | 1.53 | 2.03 | 1.00 | 1.67 | 1.33 |
|  | Cameroon | 7.41 | 7.00 | 7.80 | 7.64 | 7.24 |
|  | Congo Brazzaville | 4.40 | 3.90 | 4.89 | 4.39 | 4.41 |
|  | Cote d'Ivoire | 4.25 | 4.25 | 4.24 | 5.39 | 3.55 |
|  | Egypt | 9.95 | 9.90 | 10.00 | 9.61 | 10.10 |
|  | Ghana | 8.69 | 8.55 | 8.84 | 8.11 | 9.16 |
|  | Honduras | 15.32 | 14.68 | 16.03 | 14.91 | 15.67 |
|  | Indonesia | 8.59 | 8.78 | 8.39 | 9.83 | 7.37 |
|  | Kenya | 12.64 | 12.11 | 13.18 | 9.58 | 14.37 |
|  | Kyrgyz Republic | 0.68 | 1.01 | 0.32 | 0.35 | 0.81 |
|  | Lesotho | 24.07 | 24.72 | 23.43 | 23.03 | 24.49 |
|  | Nigeria | 2.07 | 2.29 | 1.84 | 3.09 | 1.52 |
|  | Pakistan | 9.44 | 9.07 | 9.83 | 9.15 | 9.56 |
|  | Philippines | 14.02 | 14.25 | 13.78 | 15.23 | 12.97 |
|  | Senegal | 4.26 | 4.49 | 4.02 | 4.22 | 4.28 |
|  | Yemen | 17.02 | 16.68 | 17.37 | 13.16 | 18.44 |
|  | Zambia | 6.68 | 6.67 | 6.69 | 6.95 | 6.54 |
| **Upper-middle-income countries** | | | | | | |
|  | Colombia | 27.15 | 27.34 | 26.94 | 24.86 | 32.98 |
|  | Dominican Republic | 15.23 | 15.76 | 14.66 | 15.95 | 13.15 |
|  | Gabon | 6.30 | 5.94 | 6.68 | 6.01 | 7.89 |
|  | Jordan | 12.50 | 12.12 | 12.92 | 12.92 | 10.65 |
|  | Namibia | 11.88 | 12.22 | 11.56 | 11.12 | 12.62 |
|  | Peru | 26.76 | 27.00 | 26.51 | 22.00 | 35.74 |
| **Average** | | 8.56 | 8.54 | 8.58 | 9.27 | 8.21 |
| **Median** | | 6.95 | 7.51 | 7.64 | 7.31 | 6.95 |
| **Note**: We used the de-normalized standard weight (as per the Demographic Health Survey Sampling and Household Listing Manual (ICF International 2012) as a weight in the calculation.  † The difference in the proportions of pregnancy reported unwanted in males and females was statistically significant at a 5% level of significance based on Chi-square test in all countries, except in Comoros, Malawi, Rwanda, Cote d’Ivoire and Zambia.  ‡ The difference in the proportions of pregnancy reported unwanted in urban and rural regions was statistically significant at a 5% level of significance based on Chi-square test in all countries, except in Congo Brazzaville. | | | | | | |

**Reference**

ICF International (2012) Demographic and Health Survey Sampling and Household Listing Manual. MEASURE DHS, ICF International, Calverton, Maryland, USA
